# Importance of the Quality of Annotation: Impact of Simulated Inter-Observer Variability on Deep Neural Network Automated Segmentation Model Performance

**DOI:** 10.3390/bioengineering13060691

**Published:** 2026-06-17

**Authors:** Dominic LaBella, Michaela Kop, Xuan Qi, Hunter Stecko, Baris Turkbey, Hannah Scanlon, Thomas Sanford

**Affiliations:** 1 Department of Radiation Oncology, Duke University Medical Center, Durham, NC 27705, USA; 2 John A. Burns School of Medicine, University of Hawaii, Honolulu, HI 96813, USA; 3 National Institutes of Health, Bethesda, MD 20892, USA; 4 Department of Mathematics, Duke University, Durham, NC 27705, USA; 5 Konahuanui Urology, Phoenix, AZ 85016, USA

**Keywords:** automated segmentation, prostate, MRI, deep neural network, inter-observer variability

## Abstract

Background: Deep neural network based prostate segmentation depends on manual annotations, yet the effect of annotation variability on model performance remains underexplored. Methods: Prostate contours were manually delineated by an expert clinician on 119 T2-weighted MR images from the PROSTATEx Challenge 2017 training dataset, and slice-wise synthetic radial modifications of 1–10 mm were applied to create 10 modified training datasets plus an unmodified baseline. Identical SegResNet models were trained with Auto3DSeg/MONAI and evaluated against unmodified validation and test sets using the Dice similarity coefficient (DSC). Results: Mean test DSC decreased from 0.917 for the baseline model to 0.856 at 10 mm modification. Models trained with small annotation perturbations of 1–5 mm maintained DSC values of at least 0.90, whereas performance declined significantly beyond 5 mm. Pairwise DSC agreement across modified annotations also fell as modification amplitude increased. Conclusions: Prostate segmentation models tolerated modest annotation variability but degraded substantially when variability exceeded 5 mm, underscoring the importance of annotation quality when training and benchmarking DNN-based automated segmentation models.

## 1. Introduction

Prostate cancer is the second most common malignancy and the fifth leading cause of cancer-related deaths among men worldwide [1]. Precise and consistent prostate segmentation on MRI is crucial for surgical planning, biopsy targeting, and radiotherapy planning [2,3,4,5]. However, manual prostate segmentation on MRI is not always consistent because of interobserver variability in image interpretation and boundary delineation, influenced by observer expertise, prostate morphology, and technical imaging factors [6,7,8]. Pathmanathan et al. reported a mean DSC of 0.94 among expert annotations compared with a gold standard generated via simultaneous truth and performance level estimation [9]. Langkilde et al. demonstrated greater variability among novice readers than among experienced radiologists [8], and Chen et al. highlighted considerable variability even among experts in prostate lesion segmentation [10]. Manual prostate segmentation is also time-consuming, requiring approximately 9.6 min per T2-weighted pelvic MRI scan [9].

Deep learning-based segmentation methods have emerged as a powerful solution to automate this task [6]. Modern convolutional neural networks can achieve state-of-the-art accuracy in prostate MRI segmentation, with some reports approaching expert-level performance [6]. Despite these advances, prostate boundaries can be ambiguous, particularly at the apex, and normal anatomy varies substantially, leading to differences even among expert annotations [8]. This variability in “ground truth” or more recently named “reference standard” (RS) delineations has important implications for training and evaluating segmentation models. Many deep learning algorithms are trained and validated on single-institution datasets with only one set of manual contours [6]. As a result, a model’s performance can be significantly influenced by which expert’s contours are used as the RS [11]. Current research is therefore shifting from simply maximizing Dice scores toward addressing RS variability and improving robustness across observers, imaging protocols, and institutions [6,12,13,14,15]. Recent studies have also emphasized the broader computational context for prostate cancer assessment, including intravoxel incoherent motion-based diffusion parameter estimation for computer-aided diagnosis and deep transfer learning from visually represented tabular data [16,17]. These studies illustrate that imaging inputs, model architecture, and data representation all contribute to downstream prostate cancer assessment and motivate careful evaluation of annotation quality as one component of the computational pipeline.

This study systematically evaluates how controlled synthetic modifications of RS annotations influence the robustness and accuracy of deep neural network-based prostate segmentation models.

## 2. Materials and Methods

### 2.1. Study Design

A retrospective exploratory study evaluated segmentation accuracy changes when training automated segmentation models on systematically modified prostate segmentation annotations.

### 2.2. Data and Reference Standard

**Figure 1:**
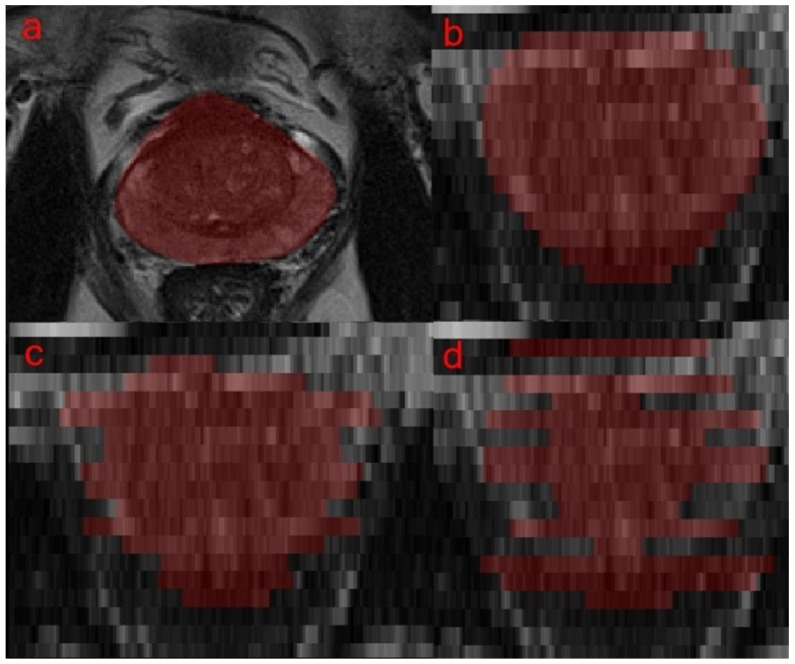
Image panels (**a**–**d**) denote prostate volume segmentations; (**a**) unmodified RS segmentation on a T2-weighted axial image; (**b**) unmodified RS segmentation on a T2-weighted coronal image; (**c**) training set segmentation with a potential 5 mm modification in either the inner or outer radial direction on each axial slice as seen on a T2-weighted coronal image; and (**d**) training set segmentation with a potential 10 mm modification in either the inner or outer radial direction on each axial slice as seen on a T2-weighted coronal image.

**Figure 2:**
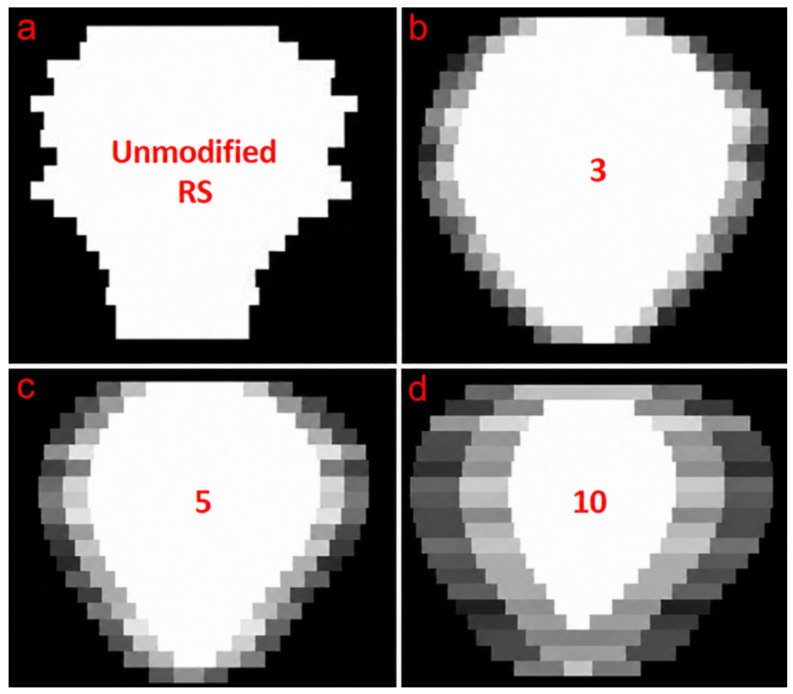
Image panels (**a**–**d**) denote a single coronal slice view of the probabilistic maps for the synthetic modifications of the prostate masks. White pixels have a probability of 1, black pixels have a probability of 0, and dark-to-light gray pixels trend from a probability of 0 to 1. (**a**) unmodified RS; (**b**) potential 3 mm modification in either the inner or outer radial direction; (**c**) potential 5 mm modification in either the inner or outer radial direction; and (**d**) potential 10 mm modification in either the inner or outer radial direction.

De-identified image data was collected from the PROSTATEx Challenge 2017 training data [18]. RS segmentations were delineated by a board certified urologist—who did a fellowship on prostate molecular and MR imaging at the National Cancer Institute—on a subset of the PROSTATEx training set axial T2-weighted prostate MRIs using DynaCAD v4.0 (Philips Healthcare, Best, The Netherlands) and based on ESTRO ACROP consensus guidelines for prostate target volume delineation [19]. Then, RS segmentations underwent synthetic radial modifications (1–10 mm) per axial slice, randomly adding outer margins, subtracting inner margins, or remaining unchanged (1/3 probability each) as shown in Figure 1. Ten datasets were created, each representing different variability levels (1–10 mm), alongside an unmodified baseline dataset. Institutional review board approval was not required because the study utilized only de-identified data sourced from publicly available datasets. In order to estimate the interobserver variability for each synthetic modification amplitude, a total of 20 different random modifications were made for each case for each respective synthetic modification amplitude. These synthetic modifications simulated realistic annotation variability, similar to that reported by previous variability studies [11,12]. This procedure is demonstrated in Figure 2, which shows the probabilistic map of the 20 different synthetic modifications with amplitude values of 3, 5, and 10 mm. Note that only 1 modification was included for each case in each of the respective training session’s training datasets. This decision was made because training datasets typically only include one image—label case for each respective image, and this is typically defined as the RS.

### 2.3. Data Partitions

A total of 119 T2-weighted MRI cases were selected based on practical considerations, balancing sufficient dataset size for robust model training with the availability constraints of the expert annotator. A dataset split of approximately 60% training, 20% validation, and 20% testing was chosen to ensure adequate representation and statistical power within the validation and testing cohorts.

### 2.4. Model and Training

The segmentation model utilized for this study was the SegResNet architecture, a convolutional neural network optimized for medical image segmentation tasks. The model was implemented within Auto3DSeg and MONAI framework, which supports robust training and validation protocols [20,21]. For each of the 10 modified and 1 unmodified datasets, an identical SegResNet model was trained over 300 epochs for a total of 11 model training sessions. The SegResNet models used five encoding–decoding blocks with a downsampling and upsampling configuration of [1,2,4], 32 initial filters, and a dsdepth of 4. The AdamW optimizer was used with an initial learning rate of 0.0002, and a weight decay of 0.00001. The DSC and cross-entropy weighted sum loss function was used with a batch size of 1 and images per batch of 1. Hyperparameters were identical across all training sessions to maintain consistency and minimize confounding variables. Only a single fold of training was performed, and all 11 of the model training sessions used identical unmodified RS image—label pairs in the validation set. For each model training session, validation loss was computed after irregular epoch intervals with more frequent validation evaluation towards the end of model training as specified by the Auto3DSeg data analysis. Final models were selected based on the highest DSC from validation with unmodified RS annotations (23 cases). The epoch associated with the highest DSC was recorded. All training was performed with an RTX 2070 with 8 GB of GDDR6 VRAM and an i7-7770K CPU with 32 GB of available RAM.

### 2.5. Evaluation

Dice Similarity Coefficient (DSC) assessed model predictions from each modified training cohort’s respective model against unmodified validation/testing RS sets (24 cases). The pairwise DSC average was used to assess the consistency across the multiple synthetically modified segmentations for each training dataset. A two-sample t-test was performed to assess whether there was a statistically significant difference between the average DSC of the test set from the baseline (unmodified) automated segmentation model and the DSC values obtained from the test sets corresponding to each modified training dataset.

## 3. Results

### 3.1. Data

A total of 119 T2-weighted prostate MRI and their respective expert annotated RS labels were included in the study. Training comprised 72 cases per modified dataset, with validation and testing sets using 23 and 24 unmodified RS cases, respectively. Clinical and demographic data were unavailable for the selected cases [18].

### 3.2. Model Performance

**Figure 3:**
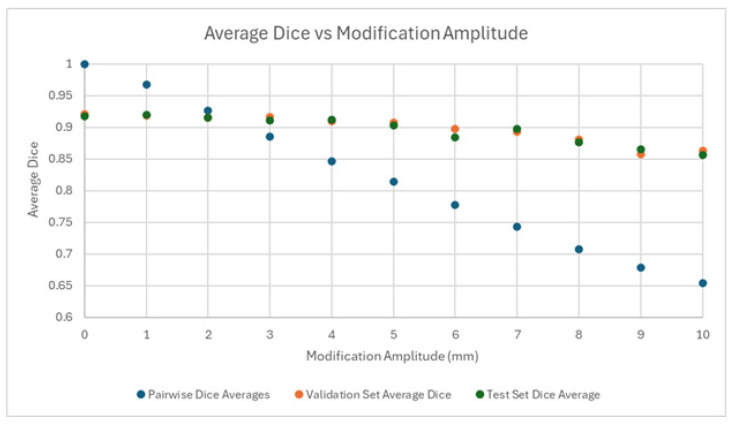
Scatterplot showing the relationship between the DSC of the automated segmentation model’s test and validation set predictions as a function of the amplitude of the respective training sets’ inner and outer radial margin axial-based random modifications. The pairwise DSC is reported as well for comparison.

Segmentation accuracy (DSC) demonstrated a downward trend as RS variability amplitude increased (baseline 0.917 ± 0.033, down to 0.856 ± 0.054 at 10 mm), as shown in Table 1 and Figure 3. Models trained with small modifications (1–5 mm) showed minimal performance reductions (DSC ≥ 0.90, *p* > 0.10 compared to baseline). Larger modifications (6–10 mm) resulted in statistically significant accuracy declines (*p* < 0.02) when compared to the baseline performance of the model trained on the 72 unmodified RS cases.

### 3.3. Pairwise Segmentation Agreement Analysis

To assess the synthetic interobserver variability as a function of modification amplitude, we calculated pairwise DSC averages across the 20 modified segmentations for each synthetic modification amplitude as shown in Table 1. Pairwise DSC consistency among modified datasets significantly declined from 0.967 ± 0.010 at 1 mm to 0.654 ± 0.043 at 10 mm (*p* < 0.0001, two-sample *t*-test).

## 4. Discussion

The purpose of this study was to investigate the influence of random variation in RS segmentations on the performance of deep learning prostate segmentation models. Accurate prostate segmentation on MRI is critical for prostate cancer management but is complicated by inherent interobserver variability in manual annotation. Random radial modifications of varying amplitudes (1–10 mm) were introduced into expert manual prostate segmentations to simulate clinically realistic variability. SegResNet models trained on these modified datasets were assessed against an unmodified testing set, revealing a significant decrease in performance only when variability exceeded 5 mm, corresponding to an intra-observer pairwise DSC of less than 0.81.

These findings suggest that highly time-consuming, perfectly precise manual annotations may not always be necessary for training robust automated segmentation models. Segmentation performance remained statistically similar despite modest synthetic variability (1–5 mm), which indicates that minor contour differences representative of typical interobserver variability do not meaningfully degrade segmentation accuracy. The identified ≤ 5 mm threshold provides practical guidance, suggesting that extensive effort to eliminate every small contour difference may yield limited returns for model performance. Similar conclusions have been reported in prior uncertainty-quantification studies, reinforcing the idea that moderate variability could improve model generalizability [11,12].

Controlled synthetic variability may also serve as a practical data augmentation strategy for enhancing model robustness [22]. This approach could reduce the annotation burden associated with large training datasets and make AI model development more feasible in clinical settings [23,24].

Key limitations of this study include first, the relatively small sample size, which was constrained by expert annotator availability. Second, the synthetic modifications were limited to simple radial shrinkage or expansion and did not account for more complex clinically realistic variations such as shape irregularities or intensity-related boundary effects. Future studies should evaluate synthetic variability techniques across other anatomical structures, larger datasets, and additional imaging modalities to better define the practical relevance of synthetic variability as a training augmentation strategy. Third, all experiments used SegResNet within MONAI, and future studies should compare nnU-Net, transformer-based, and ensemble segmentation architectures to determine whether the observed tolerance threshold generalizes across model families [25,26,27,28,29]. Finally, because RS annotations were generated by one expert annotator, multi-annotator consensus, label ensembling, and annotator-level uncertainty estimation could not be performed in the current dataset [12,30]. Future work could integrate uncertainty-aware segmentation, multimodal pre-training, graph attention, and other data-driven robustness-learning methods to evaluate model stability under annotation noise without assuming a single deterministic reference standard.

## 5. Conclusions

Controlled synthetic annotation variability of up to 5 mm did not meaningfully reduce prostate MRI segmentation performance, whereas variability above 5 mm produced statistically significant degradation. These findings suggest that moderate contour variability may be tolerable during model development, but annotation quality remains a critical factor when training and benchmarking DNN-based automated segmentation models.

## Figures and Tables

**Table 1:** Effect of annotation modification amplitude on segmentation model performance and pairwise similarity.

Modification Amplitude (mm)	Pairwise DSC Averages (Std Dev)	Validation Set Average DSC (Std Dev)	Best Epoch	Test Set Average DSC (Std Dev)	Testing Set *t*-Test *p*-Value
0	1.000 (0)	0.9241 (0.014)	257	0.917 (0.033)	1.000
1	0.967 (0.010)	0.9188 (0.015)	252	0.9194 (0.023)	0.7714
2	0.926 (0.013)	0.915 (0.019)	290	0.9157 (0.027)	0.8819
3	0.885 (0.018)	0.9159 (0.018)	234	0.9106 (0.039)	0.5424
4	0.846 (0.024)	0.9095 (0.020)	246	0.9115 (0.031)	0.5547
5	0.814 (0.029)	0.907 (0.023)	240	0.9025 (0.027)	0.1025
6	0.778 (0.032)	0.8971 (0.017)	246	0.8839 (0.037)	0.0020
7	0.743 (0.037)	0.8927 (0.029)	152	0.8971 (0.021)	0.0164
8	0.708 (0.040)	0.8813 (0.032)	240	0.8764 (0.048)	0.0013
9	0.679 (0.043)	0.8574 (0.033)	168	0.8649 (0.040)	<0.0001
10	0.654 (0.043)	0.8626 (0.043)	160	0.8561 (0.054)	<0.0001

This table presents the pairwise average DSC for each of the 20 random synthetic modifications for each modification amplitude. It also presents the average DSC of the validation and test sets, as well as the best epoch for each model, for each respective modification amplitude training set. The *p*-values represent the two-sample t-test evaluation of the baseline test set average DSC compared to the respective modification amplitude’s testing set DSC.

## Data Availability

The imaging data used in this study are publicly available from The Cancer Imaging Archive (TCIA), SPIE-AAPM PROSTATEx Challenge Data (Version 2), https://doi.org/10.7937/K9TCIA.2017.MURS5CL (accessed on 24 April 2026), available at https://www.cancerimagingarchive.net/collection/prostatex/ (accessed on 24 April 2026).

## Abbreviations

The following abbreviations are used in this manuscript:

AI	Artificial intelligence
Auto3DSeg	Automated three-dimensional segmentation
CPU	Central processing unit
DNN	Deep neural network
DSC	Dice similarity coefficient
ESTRO ACROP	European Society for Radiotherapy and Oncology Advisory Committee on Radiation Oncology Practice
GDDR6	Graphics double data rate 6
MONAI	Medical Open Network for Artificial Intelligence
MR	Magnetic resonance
MRI	Magnetic resonance imaging
RAM	Random-access memory
RS	Reference standard
SegResNet	Segmentation residual network
TCIA	The Cancer Imaging Archive
VRAM	Video random-access memory
